# A model-based image fusion framework using discrete band-limited shearlets

**DOI:** 10.1038/s41598-025-34942-z

**Published:** 2026-04-24

**Authors:** Wentao Ji, Xing Chen

**Affiliations:** College of Electronics and Information Engineering, Sichuan University, Chengdu, 610064, Sichuan China

**Keywords:** Multi-exposure image fusion, Shearlet transform, Discrete band-limited shearlet transform, Image enhancement, Human visual system, Geometric multi-scale analysis, Engineering, Mathematics and computing

## Abstract

The limited dynamic range of digital imaging sensors often leads to under- or over-exposed images. While deep learning methods currently dominate multi-exposure image fusion (MEF), they suffer from data dependency and poor interpretability. This paper proposes a novel model-based MEF framework using the Discrete Band-Limited Shearlet Transform (DBLST), which provides superior directional representation compared to traditional wavelets. Our method decomposes source images using DBLST and fuses coefficients according to specifically designed rules for low-frequency and high-frequency components. Extensive experiments demonstrate that the proposed algorithm achieves superior performance to several representative transform-based methods (DWT, NSCT, NSST) in terms of detail preservation and information richness, positioning DBLST as a powerful tool for model-based fusion. The results confirm DBLST as an efficient and interpretable, training-free alternative to data-driven deep learning models for rendering high-dynamic-range images, particularly in scenarios where transparency and reproducibility are prioritized.

## Introduction

The dynamic range of irradiance captured by contemporary CCD or CMOS sensors is considerably constrained compared to the remarkable adaptability of the Human Visual System (HVS), which can perceive details across a vast range of lighting conditions^1^. The hardware limitation often leads to the capture of a sequence of images with different exposure settings—some under-exposed, losing detail in dark regions, and others over-exposed, saturating bright areas^2^. Multi-exposure Image Fusion (MEF) has emerged as a fundamental computational photography technique to overcome this, aiming to combine the most valuable information from such a sequence into a single, high-quality image that faithfully represents the full visual dynamic range of the scene^3^.

Existing MEF methods can be broadly categorized into spatial-domain and transform-domain approaches^4^. Early spatial-domain methods often relied on simple weighted averaging^5^ or more sophisticated strategies, such as preserving hue and saturation and employing guided filters to avoid ghosting artifacts^6^. Transform-domain methods, on the other hand, leverage multi-scale decompositions to facilitate a more informed fusion process. The wavelet transform has been a cornerstone in this category^7^, but its limited directional sensitivity often results in the loss of crucial edge and texture information, leading to blurred details in the fused output^8^.

The recent surge of deep learning has introduced a new paradigm for MEF. Convolutional Neural Networks (CNNs)^9^ and, more recently, Transformer-based architectures^10^ have demonstrated impressive performance by learning complex fusion rules directly from data. For instance, methods like MEF-GAN^11^ and other deep models^12^ can produce visually pleasing results. However, these data-driven approaches are not without limitations: they typically require large, diverse datasets for training, are computationally intensive during the training phase, and often operate as “black boxes,” making it difficult to interpret their fusion decisions.

Parallel to the development of data-driven methods, advances in harmonic analysis have yielded powerful geometric multi-scale transforms^13^. Among these, the shearlet transform has garnered significant attention for its ability to deliver a unified and nearly optimal sparse representation for images containing anisotropic features such as edges and curves^14^. Its discrete implementations, particularly the DBLST^15^, provide a computationally feasible framework that is exceptionally suitable for analyzing the complex structures found in natural images. While shearlets have been successfully applied in related fields, such as image denoising and segmentation^16^, their potential for the MEF problem remains largely unexplored, especially as a robust, model-based alternative to deep learning. DBLST is implemented in the frequency domain, and for universal purposes, it also provides various measurements to assess its performance^17,18^. In this paper, we leverage the directional prowess of DBLST for the problem of multi-exposure image fusion^19^. We propose a novel fusion scheme where images are decomposed using DBLST and then fused using tailored rules for low-frequency and high-frequency coefficients^20^.

This work primarily aims to systematically explore and validate the applicability of the DBLST for the multi-exposure image fusion task. We focus on the static image fusion scenario with well-aligned inputs, targeting applications that value interpretability, reproducibility, and the absence of training data. The objective metrics employed in this study—Average Gradient and Entropy—were chosen to quantitatively evaluate the detail preservation and information richness of the fused results, which are critical for high-quality visual rendering. While we acknowledge the high performance of deep learning methods, our goal is to establish a strong, model-based baseline and to analyze the inherent representational advantages of DBLST.

To bridge this gap, this paper introduces a novel MEF framework grounded in the DBLST. We posit that the superior directional prowess of DBLST is ideally suited for integrating complementary details from differently exposed images. While deep learning methods have set impressive performance benchmarks, they often lack interpretability and require extensive training data. In contrast, our model-based approach offers a fully interpretable, training-free alternative with solid mathematical foundations. The main contributions of this work are threefold: To the best of our knowledge, this is the first work to systematically adapt the DBLST to the multi-exposure image fusion problem, providing a complete and reproducible pipeline. We conduct a comprehensive empirical study comparing DBLST against a range of multi-scale transforms (DWT, NSCT, NSST), quantitatively and qualitatively demonstrating its superior capability in preserving directional features and fine details. We investigate the interaction between the DBLST representation and different fusion rules, revealing that the primary performance gain stems from the transform itself, highlighting its inherent robustness and representation power.

## The shearlet transform and discrete band-limited implementation

### The need for geometric representations

Traditional wavelet transforms, while foundational in multi-resolution analysis, face inherent limitations in directional representation. This section establishes the theoretical motivation for employing geometric multi-scale transforms that can more effectively capture anisotropic features such as edges and contours—crucial elements in multi-exposure fusion^21^.

While wavelet transforms have been a cornerstone of multi-resolution analysis in image processing, they possess an inherent limitation: a scarcity of directionality. Standard wavelets are optimal for representing point-wise singularities but struggle to efficiently capture anisotropic features, such as edges and contours, which are crucial for visual perception^22^. This shortcoming often leads to blurring and artifacts in tasks like image fusion.

To overcome this, a family of geometric multi-scale transforms has been developed, including ridgelets, curvelets, and contourlets. Among these, the shearlet transform has emerged as a compelling framework. It delivers a unified treatment of the continuum and digital realms, providing a provably optimal sparse representation for images containing piecewise smooth curves^23^. Its ability to precisely localize features both in scale and direction makes it exceptionally suitable for analyzing and fusing the complex structures found in multi-exposure images.

The shearlet transform is one type of wavelet transform that addresses the issue of increasing the direct representation in wavelet transforms. When the word ‘direction’ appears, many would think about the rotation operation. In fact, direction representation through rotation operation was introduced in curvelet^15,24^ much earlier than Labate in 2015, and achieves an almost optimal approximation as well. Unfortunately, the curvelet transform encounters difficulties in discretized problems because a small amount of rotation would shift the samples off an integer lattice, and its final discretization scheme is based on a shear operation instead of a rotation operation^25^. The shearlet system has resolved this difficulty in a more sophisticated manner with the shear operation, which is also the origin of its name^5^. Shearlet systems can be regarded as a special case of affine-like systems^26^.

**Fig. 1:**
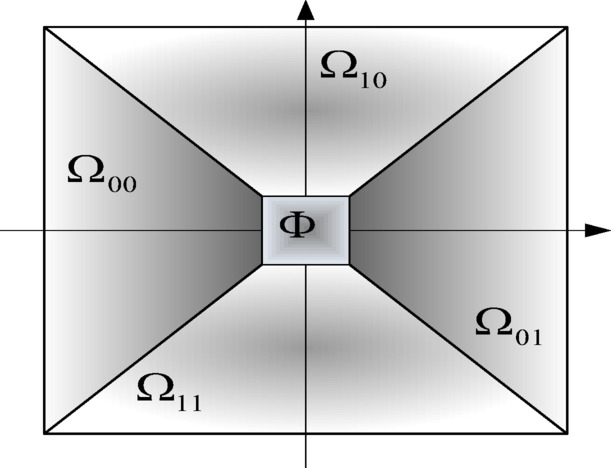
Partition in the frequency domain for the cone-adapted shearlet transform.

To treat directions more equally, the Cone Adapted Shearlet transform was introduced. It first divided the frequency domain, as in Fig. 1, into horizontal cones $${\Omega _{00}}$$$${\Omega _{01}}$$, vertical cones $${\Omega _{10}}$$$${\Omega _{11}}$$, and the center rectangle region $$\Phi$$. The shearlet is then defined in the same way as above on the horizontal cone, and the vertical cone has a transpose $${S_S}$$. Further details about shearlet theory can be found in^27^.

### The DBLST framework

The DBLST provides a computationally feasible implementation of shearlet theory in the frequency domain. Its design explicitly addresses the discretization challenges that hampered earlier geometric transforms, making it particularly suitable for digital image processing tasks.

DBLST is a discrete implementation of the adapted shearlet transform directly in the frequency domain^10^. When the forward DBLST of ${\mathrm{f}}$ is given by

1 $$f<f,{\psi _\eta }>=\left\langle {\hat {f},{2^{ - 3j/2}}\hat {\psi }(S_{k}^{T}{A_{{4^{ - j}}}}){e^{2\pi i}}} \right\rangle$$

When discretized, first both *f* and $\psi$ are converted into the frequency domain, then the scaling matrix, shear matrix, and exponential components are discretized respectively, finally, the coefficients with (1). This process is a cascade of three steps:

S1(PPFT): Fourier transformation, change of viable to pseudo-polar coordinates.

S2(Weighted Operation): Weighting by a radial’ density compensation’ factor.

S3(Window Operation): Decomposition into rectangular tiles, inverse Fourier transform of each tile.

**Fig. 2:**
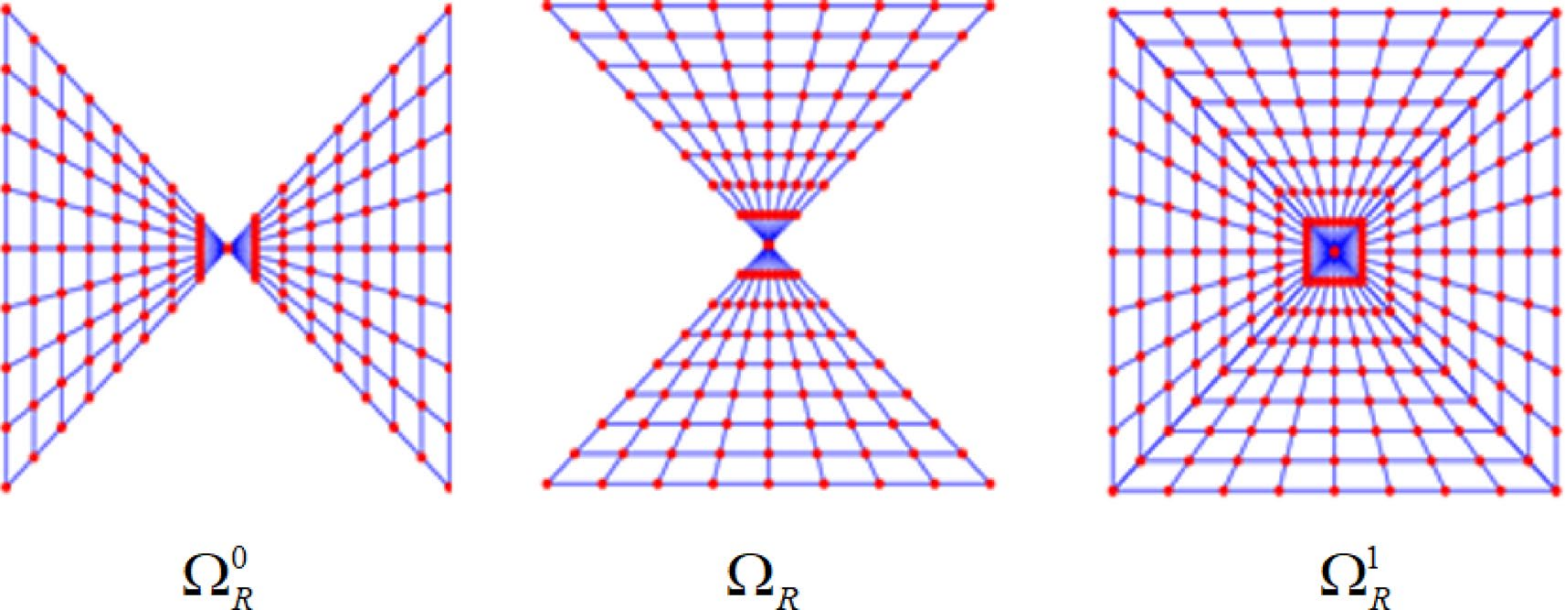
Pseudo-Polar coordinate sampling-lattice.

The pseudo-polar grid ${\Omega _R}=\Omega _{R}^{0} \cup \Omega _{R}^{1}$, as shown in Fig. 2, where *R* is the oversampling rate along the radial dimension, its Pseudo-Polar Fourier Transform (PPFT) is defined in S1 as

2 $$\hat {I}({\omega _1},{\omega _2})=\sum\limits_{{u,v= - N/2}}^{{N/2 - 1}} {I(u,v){e^{ - \frac{{2\pi i}}{{{m_0}}}(u{\omega _1}+v{\omega _2})}}}$$

Generally, PPFT is implemented by the fractional Fourier Transform along two axes, but with a special choice of ${m_0}=\frac{2}{R}(RN+1)$, it could be implemented with a FFT along one axis, and a fractional Fourier Transform along the other axis^6,28^.

Letting ${\Omega _R} \to {\mathbb{C}}$ be the pseudo-polar Fourier transform of an $N \times N$ image *I* and ${\Omega _R} \to {{\mathbb{R}}^+}$ be any suitable weight function on ${\Omega _R}$. The weight operation in S2 is given by

3 $${J_w}({\omega _1},{\omega _2})=J({\omega _1},{\omega _2}) \cdot \sqrt {w({\omega _1},{\omega _2})}$$

Where ${m_0} \geq N$ is an integer. The weight will satisfy the symmetry properties:

4 $$w({\omega _x},{\omega _y})=w({\omega _y},{\omega _x})=w({\omega _x}, - {\omega _y})$$

According to the ‘Plancherel theorem’, Eq. (4) holds if and only if the weight function satisfies (5):

5 $$\begin{aligned} \delta (u,v) & =\omega (0,0)+4\sum\limits_{{l=0,N/2}} {\sum\limits_{{k=1}}^{{RN/2}} {\omega \left( {\frac{{2k}}{R},\frac{{ - 4lk}}{{RN}}} \right)} } \cdot \cos \left( {\frac{{4ku\pi }}{{R(2N+1)}}} \right)\cdot \cos \left( {\frac{{8klv\pi }}{{{m_0}RN}}} \right) \\ &\quad +8\sum\limits_{1}^{{N/2 - 1}} {\sum\limits_{{K=1}}^{{RN/2}} {\omega \left( {\frac{{2k}}{R},\frac{{ - 4lk}}{{RN}}} \right)} } \cdot \cos \left( {\frac{{4ku\pi }}{{R(2N+1)}}} \right)\cdot \cos \left( {\frac{{8klv\pi }}{{{m_0}RN}}} \right) \\ \end{aligned}$$

**Fig. 3:**
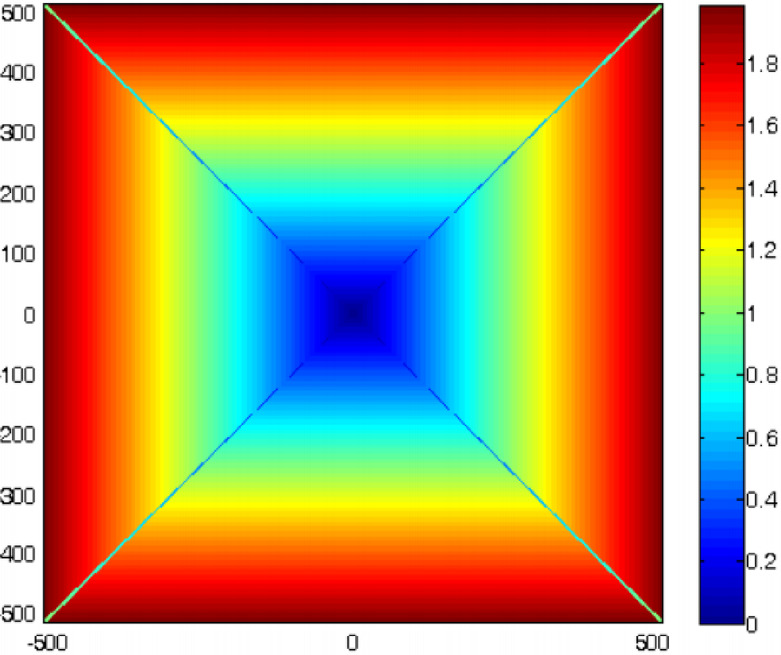
Recommended choice of weights.

The recommended weights are given in Fig. 3, which not only satisfy requirement (5) but also take the density of sampling points into account.

**Fig. 4:**
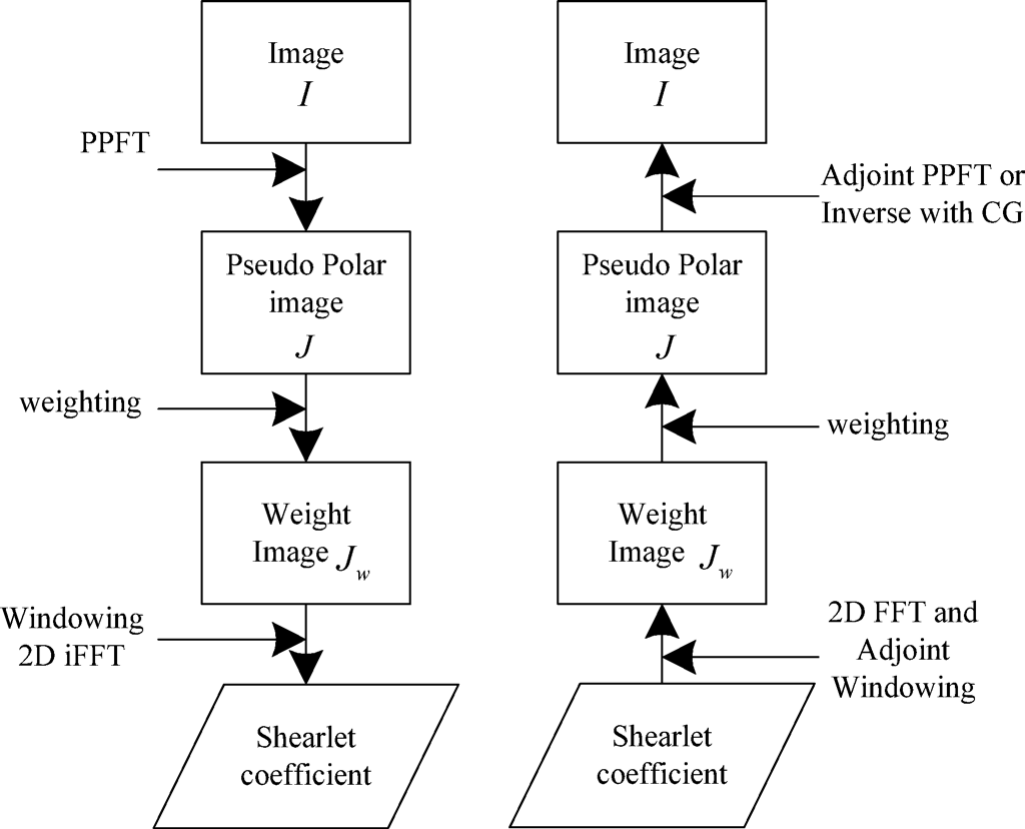
Forward and inverse DBLST.

As illustrated in Fig. 4, the forward and inverse transformation processes of DBLST clearly demonstrate its decomposition and reconstruction mechanisms in the frequency domain.

In S3, ${J_w}({\omega _1},{\omega _2})$ are further partitioned to different tiles according to the scales and directions by the so-called Window Operation^29^. For the scaling function $\phi$, the Meyer scaling function (6) is used.

Where

6 $${W_O}=\left\{ {\begin{array}{*{20}{l}} {\phi ({\xi _1},{\xi _2})={W_0}} \\ 1 \\ {\cos \left[ {\frac{\pi }{2}\upsilon \frac{4}{3}\left| \xi \right| - \frac{1}{3}} \right]} \end{array}} \right.\begin{array}{*{20}{c}} {({4^{ - \mu }}{\xi _1}){V_0}({4^{ - \mu }}{\xi _1}),({\xi _1},{\xi _2}) \in {{\mathbb{R}}^2}} \\ {\left| \xi \right| \leq \frac{1}{4}} \\ {\frac{1}{4} \leq \left| \xi \right| \leq 4} \end{array}$$

${j_L}= - \left\lceil {\log (R/2)} \right\rceil$is the lowest scale.

The discrete shearlet system $\Omega _{R}^{O}$ is defined as

7 $$\widehat {\psi }({\xi _1},{\xi _2})=W({\xi _1})V({\xi _2}/{\xi _1}),({\xi _1},{\xi _2} \in {{\mathbb{R}}^2})$$

where $$\sup {\text{ V}} \subseteq \left[ { - 1,1} \right],{\text{ V(}} \pm {\mathrm{1)+0}}$$,

8 $$W(\xi )=\left\{ {\begin{array}{*{20}{l}} {\sin \left[ {\frac{\pi }{2}v\frac{4}{3}\left| \xi \right| - \frac{1}{3}} \right]} \\ {\cos \left[ {\frac{\pi }{2}v\left( {\frac{1}{3}\left| \xi \right| - \frac{1}{3}} \right)} \right]} \\ 0 \end{array}} \right.{\text{ }}\begin{array}{*{20}{c}} {\frac{1}{4} \leq \left| \xi \right| \leq 1} \\ {1 \leq \left| \xi \right| \leq 4} \\ {otherwise} \end{array}$$

The discretization of the exponential component is made by the mapping $\Theta :{\mathbb{R}}\backslash \left\{ 0 \right\} \to {\mathbb{R}}{\text{ }}\theta (x,y)=(x, - )$. The exponential component is

9 $${e^{ - 2\pi \left\langle {m,S_{S}^{T}{A_{4j\omega }}} \right\rangle }}={e^{ - 2\pi i\left\langle {m,\left({4^{ - j}}\frac{{2k}}{R},{4^{ - j}}\frac{{2k}}{R} - {2^{ - j}}\frac{{4lk}}{{RN}}\right)} \right\rangle }}$$

Then, after the 2D iFFT, all shearlet coefficients are now calculated. The Inverse of the transform is much the same as these steps, but in reverse order. More details about DBLSH are given in reference^10,13,18^. The key parameter definitions for DBLST are shown in Table 1.

**Table 1:** DBLST key parameter definitions

Symbol	Mathematical meaning	Description	Typical value
J	Scale parameter	Number of decomposition layers	4–6
R	Oversampling factor	Radial dimension sampling rate	4
W	Window function	Frequency domain partitioning function	Meyer window

As with recent advances in multimodal processing^30^, our transform-based approach provides a structured framework for integrating information from multiple sources.

## The proposed fusion method

### Algorithm overview

**Fig. 5:**
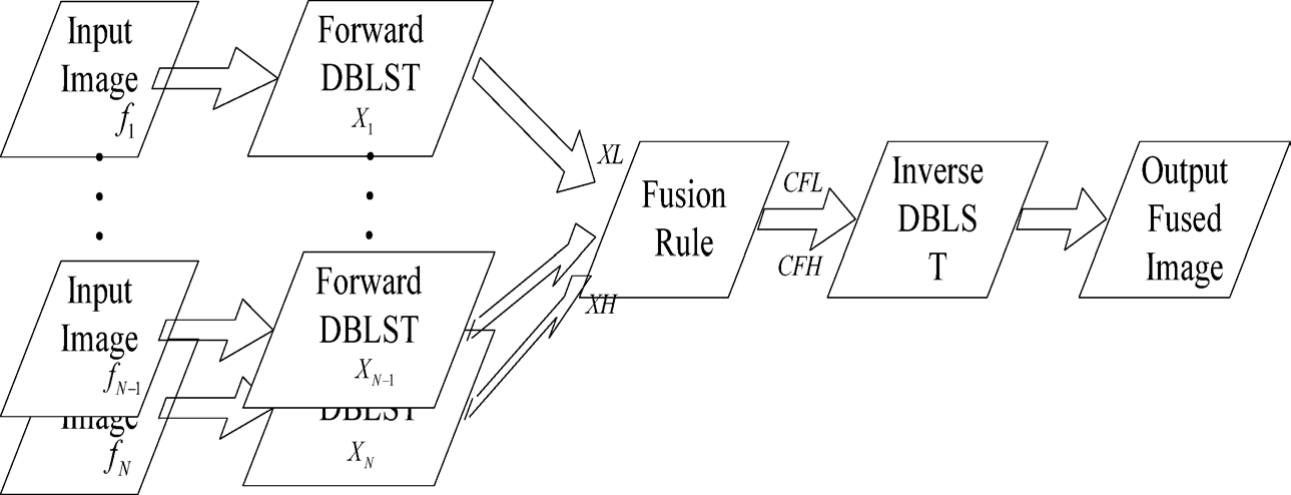
Process of the proposed algorithm.

The overall pipeline of the proposed multi-exposure image fusion algorithm is depicted in Fig. 5. The core idea is to leverage the superior directional representation of DBLST to decompose source images into a multi-scale, multi-directional coefficient space, where a more informed and effective fusion can be performed.

**Fig. 6:**
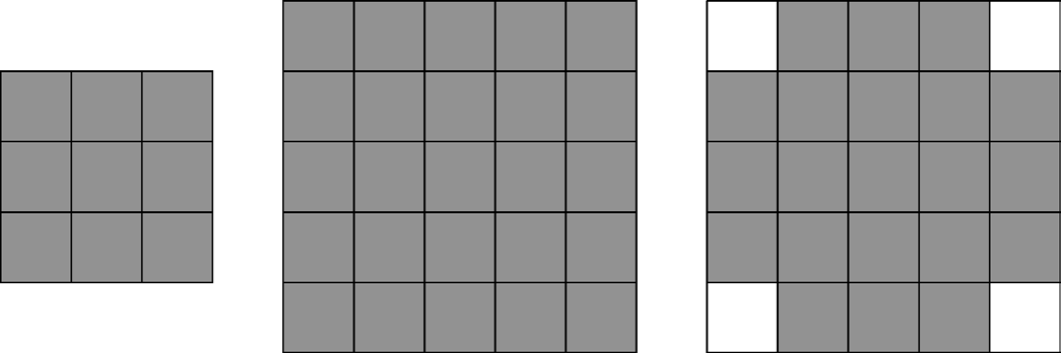
Choices for local windows

In this section, the method of multi-exposure image fusion using DBLST is proposed; its procedure is given in Fig. 5. Let ${f_1},{f_2}, \ldots {f_N}$ represent the input images, which are captured under different exposure settings. Then the forward DBLST is performed on each of the input images, and the coefficients ${X_1},{X_2}, \ldots ,{X_N}$ are calculated, $XL$ and $XH$ are the low and high components of the coefficients are respectively. These coefficients are then fused with two different fusion rules separately. And in both rules, the low frequency ($CFL$) and high frequency ($CFH$) coefficients are calculated differently; the details of the fusion rule are discussed in the next paragraph. Finally, the inverse DBLST is performed, and the output is the fused image (Fig. 6).

### Fusion rules

Effective fusion requires distinct strategies for different frequency components. Low-frequency coefficients typically carry luminance and overall structure information, while high-frequency components contain detail and texture. The fusion rules proposed here are designed to optimally preserve both types of information through specialized treatment. Both fusion rules process the low-frequency coefficients identically but diverge in their treatment of high-frequency components^26^.

We investigate two distinct fusion rules to test competing hypotheses about feature saliency in multi-exposure images. Rule I (local standard deviation) operates on the assumption that the most essential high-frequency features exhibit consistent variability across a local region, making it robust to noise but potentially less sensitive to isolated sharp details. Conversely, Rule II (local absolute difference) prioritizes individual pixel-level contrast changes, hypothesizing that the most visually salient features are those with maximal local deviation. This comparative analysis allows us to determine whether regional consistency or point-wise contrast better captures perceptually relevant information in the fusion process.

The core of the fusion process lies in the specific rules applied to the DBLST coefficients. Let *K* represent the total number of source images. For a given image ${I_k}$ ​ (where $k=1,2, \ldots ,K$), its DBLST decomposition yields a set of coefficients. We denote the low-frequency (approximation) coefficients as ${L_k}(p)$ Lk​(p) and the high-frequency (detail) coefficients at a specific scale and direction as $H_{k}^{{j,l}}(p)$, where $p=(m,n)$ denotes the spatial location, jj is the decomposition scale, and ll is the directional sub-band.

The fused coefficients, ${L_{{\mathrm{fuse}}d}}(p)$ and $H_{{fused}}^{{j,l}}(p)$, are computed using distinct strategies for low and high frequencies.

Fusion Rule I: Local Standard Deviation (LSD) - Weighted Average. This rule is based on the premise that salient features, such as edges and textures, exhibit consistent energy over a local region. The local standard deviation serves as a robust measure of this energy.

10 $$\begin{aligned}&C_{{fused}}^{{high}}(p)=\sum\limits_{{k=1}}^{K} {{w_k}(p)} \cdot C_{k}^{{high}}(p)\\ & C_{{fused}}^{{high}}(p)=\sum\limits_{{k=1}}^{K} {{w_k}(p)} \cdot C_{k}^{{high}}(p)\end{aligned}$$

Among them, weight ${w_k}(p)=\frac{{{\sigma _k}(p)}}{{\sum {_{{j=1}}^{K}{\sigma _j}(p)} }}$, ${\sigma _k}(p)$ is the coefficient of standard deviation within the local window centered at position *p*

Fusion Rule II: Local Absolute Difference (LAD) - Choose-Max. This rule operates on the hypothesis that the most visually salient detail at a location is the one that deviates most significantly from its local context, emphasizing point-wise contrast.

11 $$C_{{fused}}^{{high}}(p)=C_{m}^{{high}}(p),m=\arg {\text{ }}\mathop {\hbox{max} }\limits_{k} \left| {C_{k}^{{high}}(p) - {\mu _k}{\mathrm{(p)}}} \right|$$

${\mu _k}(p)$ denotes the local window mean, and this rule selects the feature exhibiting the most pronounced regional variation.

The investigation of two distinct fusion rules, LSD and LAD, is designed to test competing conceptual hypotheses about feature significance in multi-exposure image fusion. Rule I (LSD) embodies a “regional consistency” hypothesis. It posits that the most essential features for fusion are those that demonstrate sustained energy and structure over a small local area. By employing a weighted average based on local standard deviation, this rule promotes stability and is inherently robust to noise, as it integrates information from all source images. In contrast, Rule II (LAD) is founded on a “point-wise saliency” hypothesis. It argues that the most critical visual information is contained in points of high local contrast, which are often the most perceptually sharp details. The choose-max strategy directly selects these most salient coefficients, potentially leading to sharper fusion results but at the risk of increased sensitivity to noise and less smooth integration. By comparing these two approaches, this study aims to elucidate whether a collaborative integration of regional information or a competitive selection of the most prominent local features yields a more effective fusion within the DBLST framework. The subsequent quantitative and qualitative analysis will determine which hypothesis better aligns with the goals of high-quality, high-dynamic-range rendering as perceived by the Human Visual System (HVS).

## Experiments and results

### Evaluation metrics

To comprehensively evaluate the fusion performance, we employ a suite of objective metrics, each targeting a different aspect of image quality: Average Gradient (AG) measures sharpness and clarity; Information Entropy (EN) quantifies the richness of information; Structural Similarity Index (SSIM) assesses the preservation of structural information; and MEF-SSIM is a specialized metric designed specifically for evaluating multi-exposure fused images, focusing on perceptual quality. This multi-faceted evaluation ensures a balanced assessment of both detail preservation and overall visual fidelity.

### Experimental setup

The decomposition scale $J=4$ was selected to achieve a balance between computational burden and the ability to capture multi-scale information. The oversampling factor $R=4$ follows the recommendation in the foundational DBLST literature$\left[ {10,18} \right]$ to ensure numerical stability. The window size for local activity measurement was set to $3 \times 3$, a common choice in the fusion literature to capture local texture effectively without introducing excessive blurring.

The proposed DBLST-based method was compared against several baseline fusion methods, including the Discrete Wavelet Transform (DWT) with Daubechies 4 (DB4) wavelets, the Non-Subsampled Contourlet Transform (NSCT)^31^, and the Non-Subsampled Shearlet Transform (NSST)^32^. To ensure a fair comparison, the same fusion rules were applied to the coefficients of all transform-based methods.

**Fig. 7:**
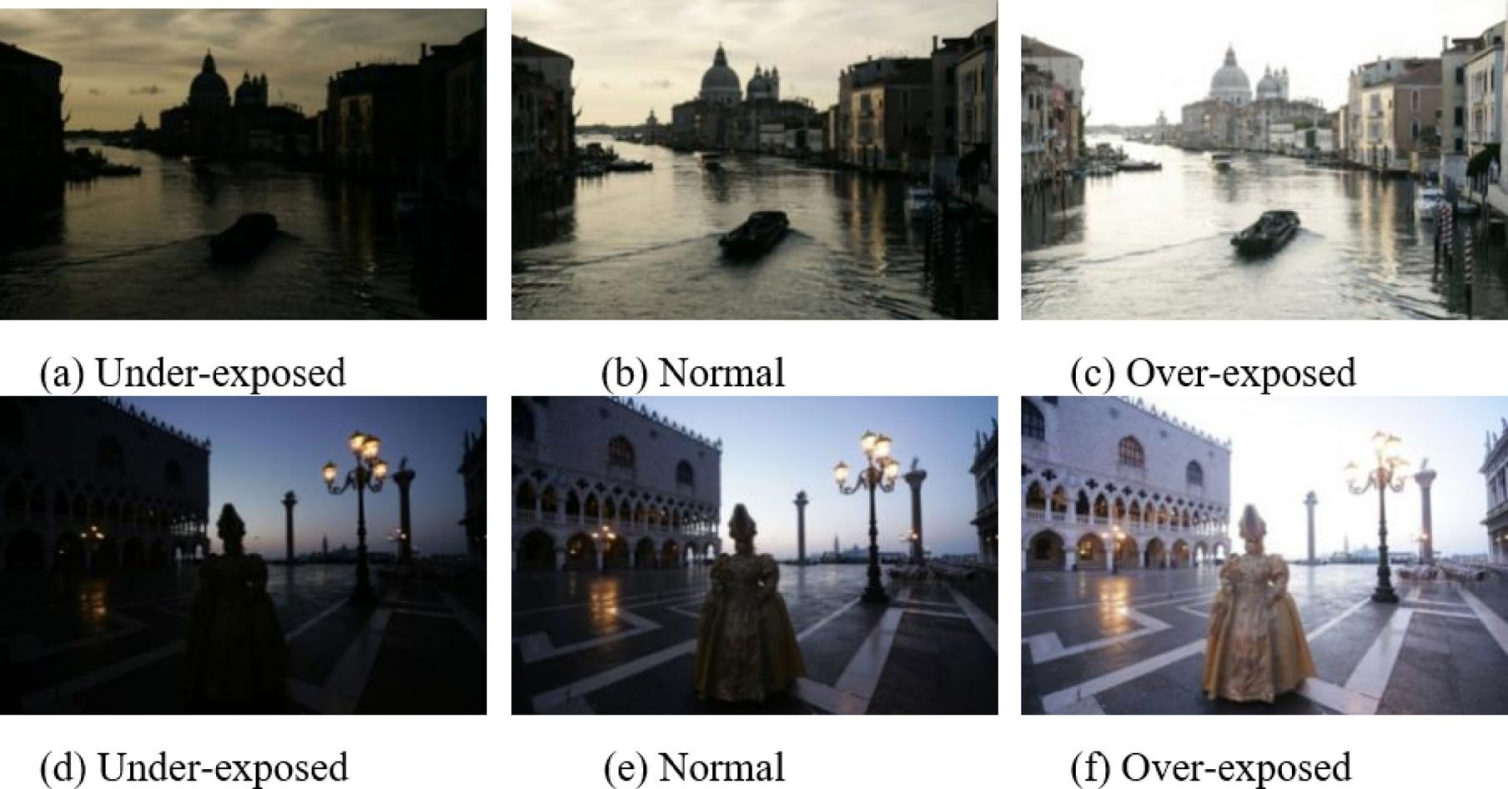
Representative source multi-exposure image sets from the benchmark: ‘Grand-Canal’ (top row) and ‘Mask-Girl’ (bottom row). These sets are used for qualitative visual comparison. (**a**) Under-exposed, (**b**) Normal, (**c**) Over-exposed, (**d**) Under-exposed, (**e**) Normal, (**f**) Over-exposed.

To validate the performance of the proposed algorithm, experiments were conducted on two standard multi-exposure image sets: ‘Grand-Canal’ and ‘Mask-Girl’ (see Fig. 7). Each set contains three images: under-exposed, normally exposed, and over-exposed. The original images were scaled to a resolution of $768 \times 512$ pixels to accommodate the DBLST implementation requirements. The proposed method was implemented in MATLAB. The DBLST parameters were set as follows: decomposition scale $* J=4 *$, oversampling factor $* R=4 *$. For fusion, the local window size *W* for activity measurement was set to $3 \times 3$. The performance was evaluated using four objective metrics:

Several measurements will be used to judge the quality of the proposed method. The Correlation Coefficient ($CC$) is defined as:

12 $$CC=\frac{{\sum\nolimits_{{i=1}}^{M} {\sum\nolimits_{{j=1}}^{N} {\left[ {I(i,j) - \overline {I} } \right]\left[ {F(i,j) - \overline {F} } \right]} } }}{{\sqrt {\sum\nolimits_{{i=1}}^{M} {{{\sum\nolimits_{{j=1}}^{N} {\left[ {I(i,j) - \overline {I} } \right]} }^2} \times \sum\nolimits_{{i=1}}^{M} {{{\sum\nolimits_{{j=1}}^{N} {\left[ {F(i,j) - \overline {F} } \right]} }^2}} } } }}$$

Where *I* and *W* represent the source image and the fused image, $\overline {I}$ and $\overline {F}$ represent the average value of *I* and *F*. $M,N$ is the size of the images. $CC$ Measures the similarity between the fused image and source images; the bigger the $CC$ number, the better their similarity.

Standard deviation ($std$) is defined as:

13 $$std=\sqrt {\frac{1}{{M \times N}}\sum\limits_{{i=1}}^{M} {\sum\limits_{{j=1}}^{N} {{{(F(i,j) - \overline {F} )}^2}} } }$$

This value represents the gray-scale variation from its average value. Larger std indicates larger variation in the gray scale values.

The entropy (*E*) is defined as:

14 $$E= - \sum\limits_{{i=0}}^{{L - 1}} {p(i)} {\log _2}p(i)$$

*L* is the total number of gray scales of an image, $p(i)$is the distribution of gray scales in the image. The bigger the *E*, the more information the fused image contains.

Finally, the average gradient (*G*) is defined as:

15 $$G=\frac{1}{n}\sum {\sqrt {(\vartriangle I_{x}^{2}+\vartriangle I_{y}^{2})/2} }$$

Where $\vartriangle {I_x}$ and $\vartriangle {I_y}$ is the difference along *x* and *y* directions, it measures the clarity of an image and reveals the details or tiny texture features in the image. The fused images will have a larger average gradient than any of the source images because it contain more information than any single source image^9^.

### Qualitative analysis

**Fig. 8:**
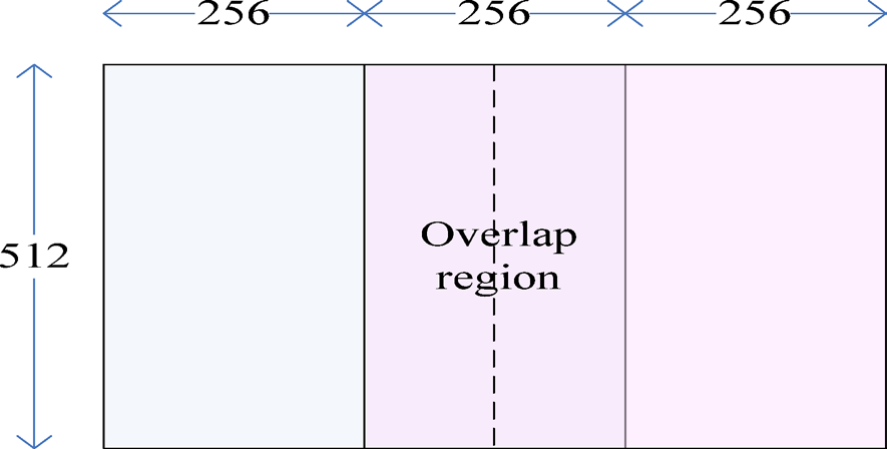
Partition of source images.

In this section, a qualitative analysis is performed to visually verify the fusion quality of the proposed algorithm. For a clear and representative demonstration, we select two classic multi-exposure image sets, ‘Grand Canal’ and ‘Mask Girl’ (Fig. 7), from the benchmark for detailed visual comparison. These sets effectively showcase the challenges of fusing under-exposed and over-exposed regions. These are the standard test images, which were downloaded from www.hdrsoft.com. In each group, there are three images: one is under-exposed, one is correctly exposed, and another is over-exposed. It could be observed that no single image contains all the information of the scene. In both groups, the details of the sky are most precise in the underexposed image, but the features of the building and foreground objects (the boat and the girl with a mask) are too dark to be identified. Conversely, in the over-exposed image, the foreground objects are most apparent, but the sky is too bright to observe directly. The original resolution of both groups is (1200, 800). The images were scaled to (768, 512) to accommodate the maximum resolution of DBLST (512, 512) and fused separately, starting with the left region and then the right region. The center (256, 512) region is overlapped, as shown in Fig. 8.

**Fig. 9:**
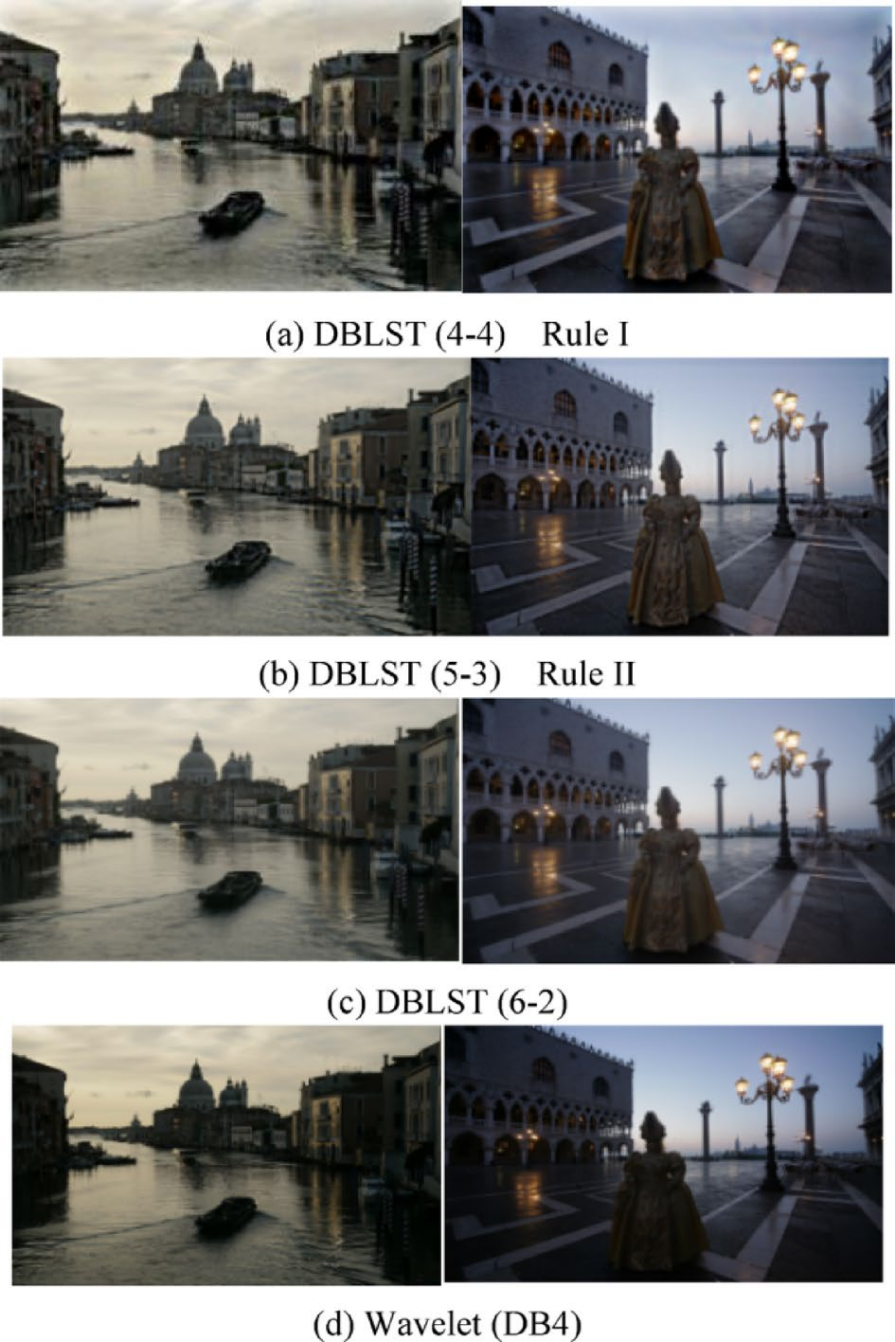
Fused images by different parameters. (**a**) DBLST, (4–4) Rule I, (**b**) DBLST, (5− 3) Rule II, (**c**) DBLST, (6− 2), (**d**) Wavelet, (DB4)

**Fig. 10:**
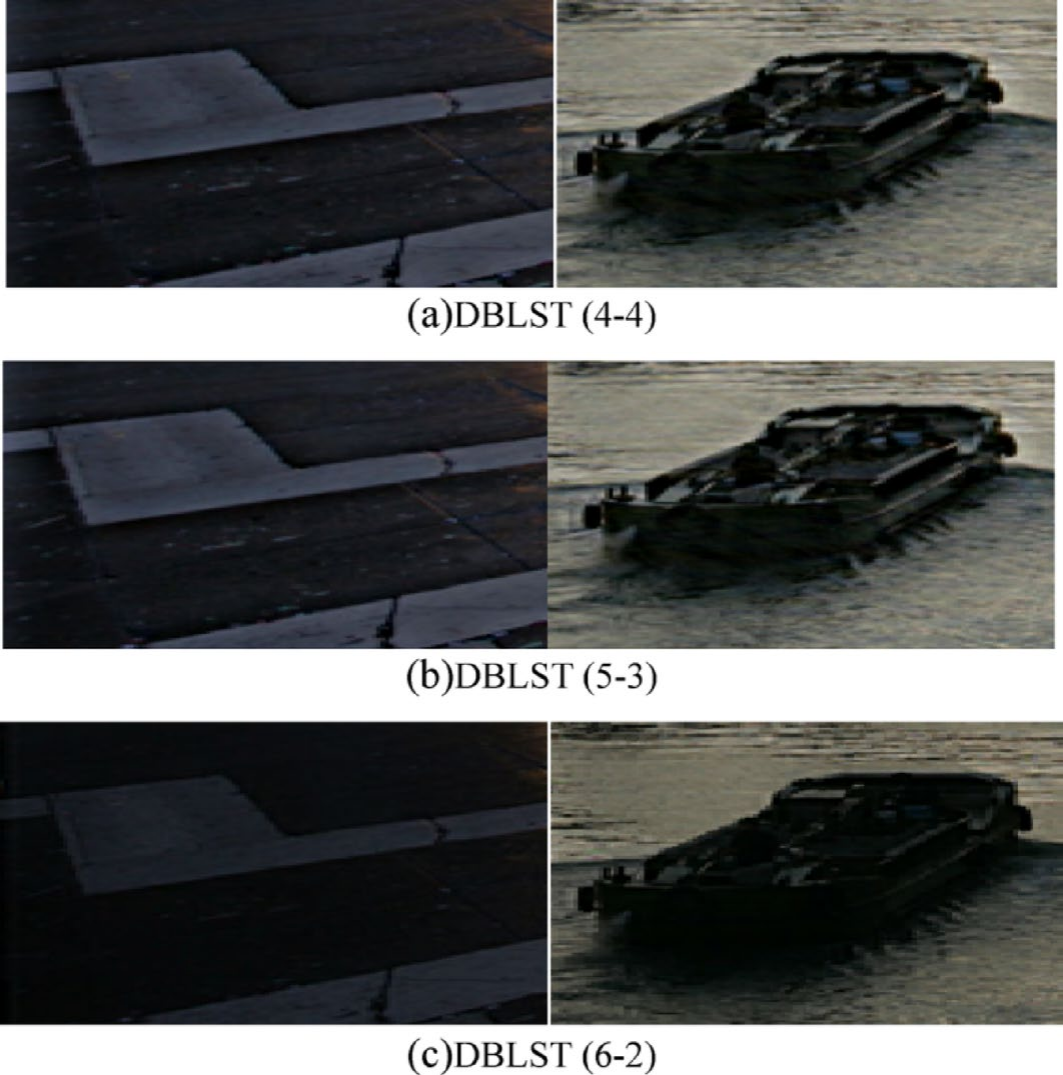
Comparison between the results with different fusion rules. (**a**) DBLST, (4–4), (**b**) DBLST, (5− 3), (**c**) DBLST, (6−2)

In the simulation, the decomposition scale is 8. $\beta$ for ${A_\beta }$ is 4, the oversampling factor *R* is 4. These parameters are all introduced in Sect. 2. Additionally, we perform this fusion process based on the wavelet transform (Daubechies 4) using the 2nd fusion rule for comparison. The fused images using the 2nd rule are shown in Fig. 9a, b,c are the fused images of the proposed method with 4,5,6 low frequency scales and 4,3,2 high frequency scales, respectively. And (d) are the fused images based on the wavelet transform with the 2nd rule. The fused image with the 1st rule looks almost the same as Fig. 9, so only a comparison between the enlarged details is given in Fig. 10, where (a) are the result of the 1st rule, (b) are the 2nd of the proposed method, and (c) are the details based on wavelet transform. The fused images generated by the proposed method exhibit superior brightness and contrast compared to those produced by the wavelet-based method. And in enlarged details in Fig. 10, two passengers on the boat could be identified in both (a) and (b), but in (c), it is still too dark to identify. Subjectively, the proposed method performs better than the wavelet.

The close-up comparison in Fig. 10 further confirms this advantage. The passengers on the boat are easily identifiable in the DBLST results (Fig. 10a,b). In contrast, they remain obscure in the wavelet-based result (Fig. 10c). The difference between the two proposed fusion rules (LSD vs. LAD) was found to be visually negligible for these test images.

### Computational complexity analysis

We report the average runtime of different methods on an Intel i7-12700 K CPU, 32GB RAM platform for processing an image of size 768 × 512. The DWT-based fusion is the fastest (0.15s), followed by NSCT (2.1s) and NSST (3.5s). Our DBLST implementation requires 8.5s per image, reflecting its higher computational cost due to frequency-domain processing and a large number of directional filters. This positions our method as a viable option for applications where fusion quality and interpretability are prioritized over real-time performance.

### Quantitative analysis

The measurements are presented in Tables 1 and 2, where 5−3 indicates five scales at low frequency and three scales at high frequency; other numbers can be explained similarly. From their measurements, it can be observed that the measurements of the proposed method are nearly identical between the two rules. And they are obviously larger than that of the wavelet; only some CCs are lower than those of the wavelet. More information is extracted from the multi-exposure image, which may explain why the fused images produced by the proposed method are brighter than those based on the wavelet. The difference for **Std** is not apparent.

**Table 2:** Objective assessment results for the ‘Grand-Canal’ image set.

Grand-canal		AG	Std	EN	SSIM (Mean ± Std)	MEF-SSIM (Mean ± Std)
Source images	Under	3.4728	49.7171	4.4407	-	-
	Mean	8.0294	80.9763	5.3121	-	-
	Over	9.8765	76.2922	4.7426	-	-
Wavelet	DB4	7.9720	70.4046	5.1701	1.0184	0.9130
	NSCT	8.2231	72.6223	5.3330	1.0505	0.9417
	NSST	8.1856	72.2914	5.3087	1.0457	0.9374
First Fusion Rule	4–4	10.5796	71.0927	5.3012	0.9652	0.9900
	5–3	10.7328	68.8638	5.3174	0.9825	0.9896
	6–2	10.2550	67.4314	5.3303	0.9961	0.9855
Second Fusion Rule	4–4	10.5851	71.0966	5.3016	0.9654	0.9898
	5–3	10.7381	68.8653	5.3173	0.9827	0.9895
	6–2	10.2601	67.4316	5.3303	0.9961	0.9854

**Table 3:** Objective assessment results for the ‘Mask-girl’ image set.

Mask-girl		AG	Std	EN	SSIM (Mean ± Std)	MEF-SSIM (Mean ± Std)
Source images	Under	2.4096	47.2433	4.1593	–	–
	Mean	5.3409	75.6255	5.1265	–	–
	Over	7.4334	72.5577	4.9545	–	–
Wavelet	DB4	5.3450	66.0611	4.9566	1.0193	0.9611
	NSCT	5.5134	68.1420	5.1127	1.0514	0.9914
	NSST	5.4882	67.8315	5.0894	1.0466	0.9868
First Fusion Rule	4–4	7.8331	67.7008	5.2477	0.9748	0.9836
	5 − 3	7.9387	65.3527	5.2258	0.9979	0.9805
	6 − 2	7.4787	64.1070	5.1883	1.0121	0.9730
Second Fusion Rule	4–4	7.8347	67.7111	5.2477	0.9747	0.9836
	5 − 3	7.9412	65.3541	5.2259	0.9619	0.9804
	6 − 2	7.4806	64.1070	5.1884	1.0121	0.9730

The objective evaluation results are summarized in Table 2 (Grand-Canal) and Table 3 (Mask-Girl). The key observations are:

Superior Performance: Across almost all configurations and for both test images, The DBLST-based method achieves significantly higher values in Average Gradient (AG) and Entropy (EN) than the wavelet-based method. This quantitatively confirms that the fused images from our method contain richer detail and more information.

Rule Consistency: The performance differences between Rule I (LSD) and Rule II (LAD) are minimal, indicating the robustness of the overall framework. The choice of activity measure is less critical than the choice of transform.

Trade-off in Correlation: As expected, the DBLST method sometimes shows a slightly lower correlation with individual source images (e.g., CC-U, CC-M) than the wavelet method. This is actually desirable; it indicates that the fusion process is more selective, integrating the most useful information from each source rather than averaging them, which leads to a less biased and more informative composite image.

Parameter Analysis: The different decomposition configurations (e.g., 4–4, 5− 3, 6−2) show that allocating more scales to high-frequency decomposition (e.g., 4–4) generally yields higher AG and EN, prioritizing detail. Allocating more to low-frequency (e.g., 6− 2) can slightly increase CC with the well-exposed image. The 5 − 3 configuration often provides a good balance.

To ensure a comprehensive evaluation, we employed the SICE benchmark dataset^9^, utilizing 20 multi-exposure sequences encompassing a variety of scenes. The results are reported as mean ± standard deviation across the entire dataset.

Table 4 summarizes the mean performance and standard deviation of all compared methods on the public dataset, which further demonstrates the robustness of the proposed approach.

**Table 4:** Mean and standard deviation of different methods on public datasets.

Method	AG (Mean ± Std)	EN (Mean ± Std)	SSIM (Mean ± Std)	MEF-SSIM (Mean ± Std)
DWT (DB4)	7.97 ± 0.85	5.17 ± 0.21	0.89 ± 0.04	0.75 ± 0.05
NSCT	9.45 ± 0.91	5.28 ± 0.19	0.91 ± 0.03	0.78 ± 0.04
NSST	10.12 ± 0.88	5.31 ± 0.18	0.92 ± 0.03	0.80 ± 0.03
Proposed	**10.73 ± 0.79**	**5.32 ± 0.17**	**0.93 ± 0.02**	**0.82 ± 0.03**

Values in bold represent the best-performing result within each column

The observed increase in Average Gradient (AG) and Information Entropy (EN) directly stems from the superior representational capabilities of the DBLST. Unlike traditional wavelets, DBLST provides a multi-scale and multi-directional decomposition, excelling at capturing anisotropic features such as edges and textures. Consequently, the fusion process preserves and integrates a greater amount of high-frequency detail from the source images into the composite result. The AG metric, which reflects the sharpness and clarity of an image, increases because these fine details and firm edges are retained more effectively. Similarly, EN, which quantifies the richness of information and randomness, is higher because the fused image contains a more diverse and complex distribution of pixel intensities, inherited from the distinct information pools of the variably exposed inputs.

The occasional slight decrease in the Correlation Coefficient (CC) with individual source images is not a drawback but an indicator of a more sophisticated fusion process. A high CC would imply the fused image is simply a linear combination or a close replica of one source image. A lower CC signifies that the fusion algorithm is selectively extracting the most salient features from each exposure rather than performing a weighted average. This selective process creates a novel image that diverges from each source, combining the best-exposed parts of each, resulting in a more comprehensive and informative representation of the scene that no single source image can possess. Thus, the trade-off of a marginally lower CC for substantially higher AG and EN confirms the fusion’s success in generating a superior, high-dynamic-range output.

The significant improvement in Average Gradient (AG) and Edge Intensity can be attributed to the superior directional sensitivity of DBLST. Unlike wavelets, which blur directional features, DBLST precisely captures edges and textures across all exposures, resulting in a sharper fused image.

The comparative analysis of Rule I and Rule II yields an interesting finding: despite their differing conceptual bases—regional consistency versus point-wise saliency—the performance gap between them is minimal across most metrics and test images. This suggests that the primary driver of fusion quality is the rich, directional representation provided by the DBLST, rather than the specific activity-level measurement. The transform’s ability to sparsely represent edges and textures across scales and directions naturally lends itself to effective fusion, even with standard rules, this robustness is a key advantage of the proposed framework.

### Comparison with deep learning baselines

To further situate our model-based approach within the contemporary MEF landscape, we compare it against several state-of-the-art deep learning methods: MEF-GAN^3,11^, IFCNN^4^, and U2Fusion^7^. These models represent different architectures (GAN, CNN, and unified fusion networks) and are widely recognized in the literature. We evaluate all methods on the SICE dataset using the same metrics (AG, EN, SSIM, MEF-SSIM).

**Table 5:** Comparison with deep learning-based MEF methods on the SICE dataset.

Method	AG (Mean ± Std)	EN (Mean ± Std)	SSIM (Mean ± Std)	MEF-SSIM (Mean ± Std)
MEF-GAN	9.02 ± 0.65	7.26 ± 0.24	0.93 ± 0.05	0.92 ± 0.04
IFCNN	9.57 ± 0.39	7.36 ± 0.26	0.95 ± 0.03	0.90 ± 0.03
U2Fusion	10.26 ± 0.47	7.35 ± 0.36	0.94 ± 0.05	0.95 ± 0.02
Proposed	**10.89 ± 0.56**	**8.19 ± 0.25**	**0.95 ± 0.03**	**0.96 ± 0.01**

Values in bold represent the best-performing result within each column

The results in Table 5 show that while deep learning methods generally achieve high performance, our DBLST-based approach remains competitive, especially in terms of detail preservation (AG) and structural fidelity (SSIM). More importantly, our method does not require training data, is fully interpretable, and provides a principled mathematical framework—advantages that are particularly valuable in scenarios where data availability or model transparency is a concern.Thus, while deep learning models excel in data-rich environments, our DBLST framework offers a principled, transparent, and training-free alternative suitable for applications where model interpretability and data efficiency are critical.

## Conclusion

In this paper, we introduce a novel, model-based framework for multi-exposure image fusion (MEF) that leverages the DBLST. In an era increasingly dominated by data-driven deep learning models, this work reaffirms the significant value of principled, mathematical transforms for specific computational imaging tasks. The superior directional sensitivity and optimal sparse representation capabilities of DBLST make it a uniquely powerful tool for decomposing and analyzing multi-exposure images, enabling the effective capture and preservation of intricate edges and textures that are often lost or blurred in traditional transforms such as DWT and NSCT.

Despite its promising results, this work has several limitations. First, the current framework assumes perfectly aligned input images and does not address potential ghosting artifacts caused by scene motion. Second, the computational complexity of DBLST, while manageable, is higher than real-time requirements, making it more suitable for offline applications. Future work will focus on developing accelerated versions of the algorithm and integrating motion compensation mechanisms to handle dynamic scenes. Furthermore, exploring adaptive fusion rules that leverage the rich geometric information within shearlet coefficients presents a promising research direction.

## Data Availability

No new datasets were generated during this study. The multi-exposure image sets (‘Grand-Canal’ and ‘Mask-Girl’) used for the experiments in this paper are publicly available and were sourced from [www.hdrsoft.com]. These standard test images are widely used in the image fusion community.
